# Semaphorin 6A Attenuates the Migration Capability of Lung Cancer Cells via the NRF2/HMOX1 Axis

**DOI:** 10.1038/s41598-019-49874-8

**Published:** 2019-09-16

**Authors:** Li-Han Chen, Che-Yu Liao, Liang-Chuan Lai, Mong-Hsun Tsai, Eric Y. Chuang

**Affiliations:** 1 Graduate Institute of Biomedical Electronics and Bioinformatics, National Taiwan University, Taipei, Taiwan; 2 Institute of Biotechnology, National Taiwan University, Taipei, Taiwan; 3 Institute of Physiology, National Taiwan University, Taipei, Taiwan; 4 Genome and Systems Biology Degree Program, National Taiwan University, Taipei, Taiwan; 5 Center for Biotechnology, National Taiwan University, Taipei, Taiwan; 6 Bioinformatics and Biostatistics Core, Center of Genomic Medicine, National Taiwan University, Taipei, Taiwan; 7 Agricultural Biotechnology Research Center, Academia Sinica, Taipei, Taiwan; 8 School of Chinese Medicine, China Medical University, Taichung, Taiwan; 9 Biomedical Technology and Device Research Laboratories, Industrial Technology Research Institute, Hsinchu, Taiwan

**Keywords:** Lung cancer, Metastasis

## Abstract

Cell migration is a fundamental feature of cancer recurrence. Since recurrence is correlated with high mortality in lung cancer, it follows that reducing cell migration would decrease recurrence and increase survival rates. Semaphorin-6A (SEMA6A), a protein initially known as a regulator of axonal guidance, is down-regulated in lung cancer tissue, and low levels of SEMA6A are associated with cancer recurrence. Thus, we hypothesized that SEMA6A could suppress cancer cell migration. In this study, we found that the migration capability of H1299 lung cancer cells decreased with SEMA6A overexpression, while it increased with SEMA6A silencing. Moreover, silencing of the cellular homeostasis protein Heme-oxygenase-1 (HMOX1) and/or the transcription factor Nuclear Factor, Erythroid-2-Like-2 (NRF2) reversed the migration-suppressing effect of SEMA6A and the SEMA6A-driven alterations in expression of urokinase insulin-like-growth-factor-binding-protein-3, Matrix metalloproteinase (MMP)-1, and MMP9, the downstream effectors of HMOX1. Taken together, these results demonstrate that SEMA6A is a potential suppressor of cancer migration that functions through the NRF2/HMOX1 axis. Our results explain why low SEMA6A is linked to high recurrence in the clinical setting and suggest that SEMA6A could be useful as a biomarker or target in lung cancer therapy.

## Introduction

Increases in cell migration, invasion, and metastasis are hallmarks of malignancy^1^. Metastasis has also been reported as a dominant cause of recurrence and a major obstacle to success in the treatment of cancer^2,3^. In the United States, lung cancer patients’ 5-year survival rate is lower in the regional stage (28%) than in the localized stage (55%). Moreover, the 5-year survival rate is only 4% in distant-stage patients^4^. Since mortality steeply increases when metastasis occurs in lung cancer patients, investigation of the genes involved in suppression of metastasis should make a significant contribution to lung cancer therapy.

Semaphorins were initially reported to regulate short-range axonal guidance. More recently, the role of semaphorins in cell migration, carcinogenesis, and cancer metastasis has been addressed in several studies. For example, semaphorins 3A, 3B, and 3F reduced growth and metastasis in melanoma, breast cancer, and colorectal cancer, respectively^5–7^. Similar to these tumor suppressor semaphorins, semaphorin 6A (SEMA6A) significantly inhibited the growth of lung cancer cells when losing either SEMA domain or whole extracellular region^8^. Moreover, we observed that SEMA6A was down-regulated in lung cancer tissue compared to its adjacent normal tissue^9^. However, the role of SEMA6A in cancer progression has been examined in only a few studies^9,10^.

SEMA6A is a single-pass transmembrane protein^11–14^ and can act as either a ligand or a receptor^15–18^. The extracellular region of SEMA6A controls the guidance of axons in embryonic development, and the intracellular region has been revealed to guide the navigation of axons by reorganizing the neuronal cytoskeleton^18^. Since both axonal guidance and metastasis are associated with the alteration of cell morphology and cell movement, SEMA6A may also regulate the migration of lung cancer cells. Although Chien *et al*. (2017) linked low expression of SEMA6A to recurrence by analyzing the data from 504 papillary thyroid cancer patients^19^, the effect of SEMA6A on migration in cancer cells has not been investigated.

Based on our current knowledge of SEMA6A, we hypothesized that SEMA6A can reduce the migration of lung cancer cells. To test this hypothesis, we compared the migration capability of H1299 lung cancer cells with and without SEMA6A overexpression. Microarray data from H1299 cells overexpressing SEMA6A versus control cells were also analyzed to identify genes potentially involved in the SEMA6A-related signaling pathway of migration. Finally, gene regulation techniques were used to confirm the effects of this pathway.

## Results

### SEMA6A’s regulation of H1299 cell migration requires its cytosolic region

**Figure 1:**
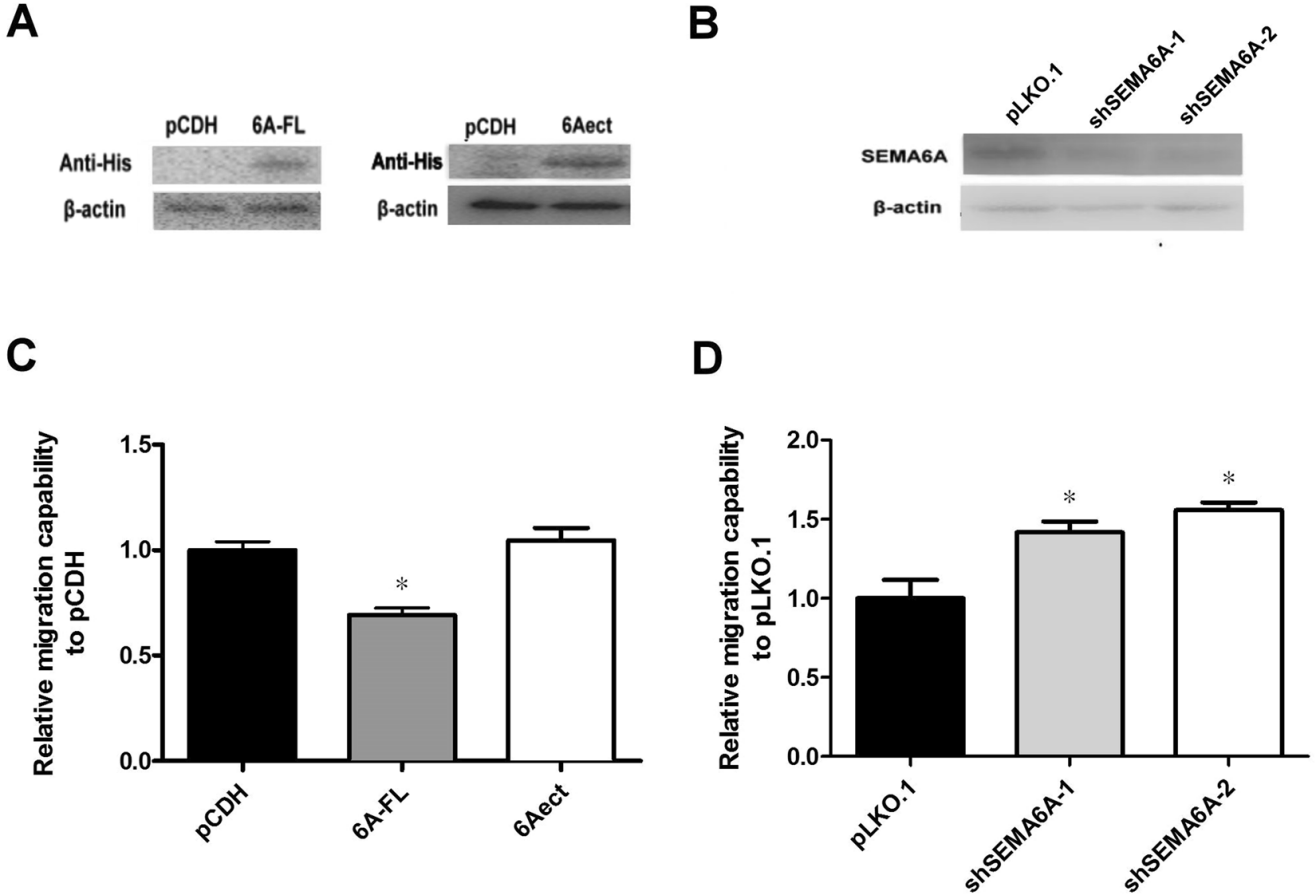
Effect of SEMA6A expression in H1299 cells on migration by transwell assay. (**A**) Western blot of His-tagged 6A-FL and 6Aect. (**B**) Western blot of SEMA6A. (**C**) Migration capability of 6A-FL- and 6Aect-overexpressing H1299 cells. (**D**) Migration capability of H1299 cells with SEMA6A silenced by two different shRNAs. *Statistical significance compared to empty vector-transfected H1299 cells at *p* < 0.05, n = 3.

For investigating the effect of SEMA6A on lung cancer cell migration, we overexpressed either the full-length DNA constructs of SEMA6A (6A-FL) or the DNA construct of the ectodomain (6Aect, residues 1-704) (Fig. 1A), silenced endogenous SEMA6A (Fig. 1B) in H1299 cells, and performed a transwell assay. The migration capability of 6A-FL-overexpressing cells decreased by approximately 35% compared to the empty vector-transfected control cells (Fig. 1C). On the other hand, SEMA6A-silenced H1299 cells had a higher migration rate than empty vector-transfected cells (Fig. 1D). Moreover, there was no difference between 6Aect-overexpressing cells and control cells (Fig. 1C). Since the 6Aect construct, composed of only the transmembrane region and extracellular ectodomain of SEMA6A, could not decrease cell migration, the cytosolic region of SEMA6A must be necessary for the migration-reducing effect.

### Heme oxygenase 1 (HMOX1) is induced in SEMA6A-overexpressing H1299 cells

**Figure 2:**
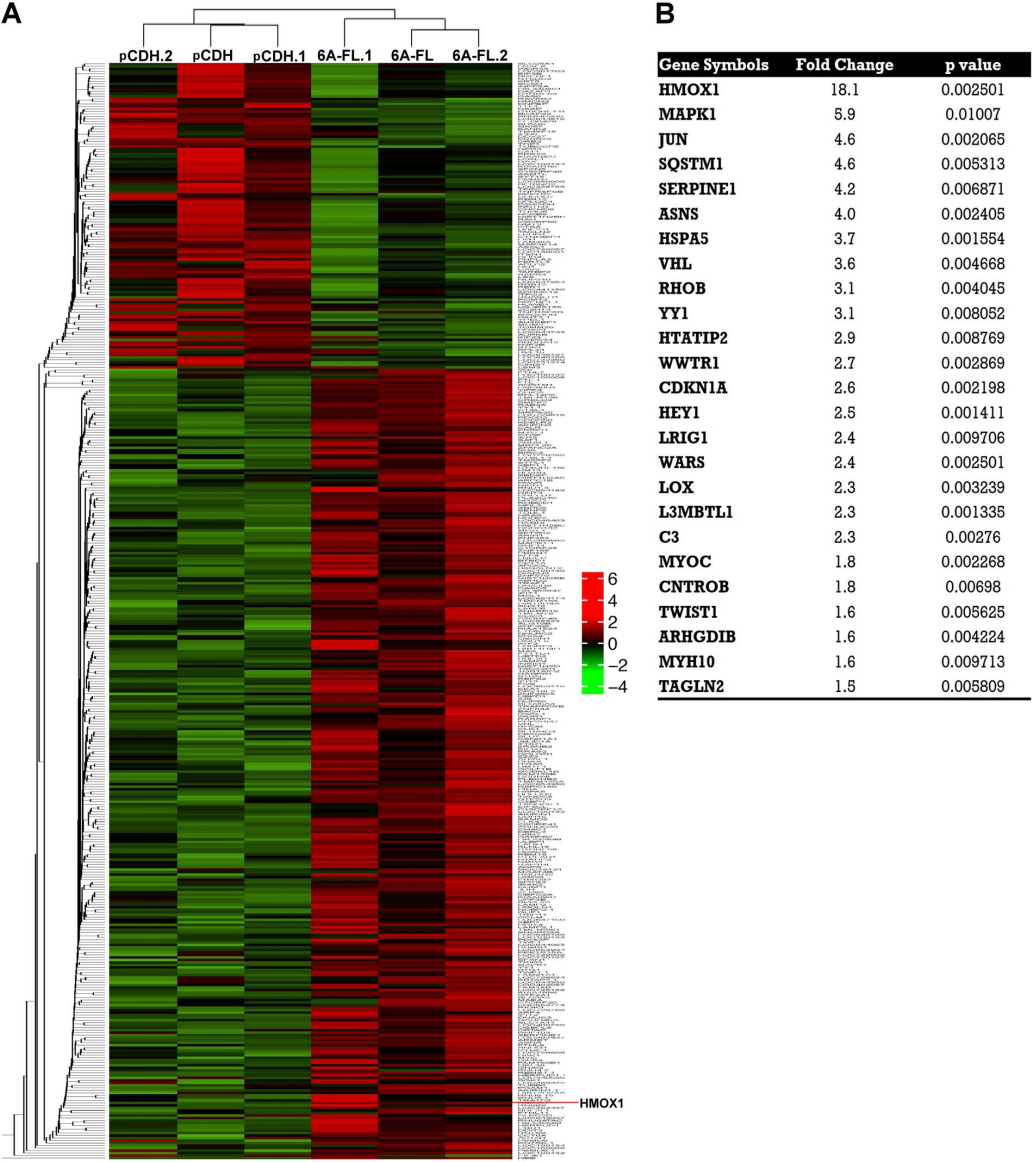
Heatmap and hierarchical clustering of microarray profiles from the pCDH-transfected (pCDH, pCDH.1, pCDH.2) and 6A-FL-overexpressing (6A-FL, 6A-FL.1, 6A-FL.2) H1299 cells (**A**), and the migration-related genes fold change >1.5 in 6A-FL-overexpressing H1299 cells (**B**).

After SEMA6A was demonstrated to modulate lung cancer cell migration, we wanted to identify the genes downstream of SEMA6A in the migration inhibition pathway. Microarray data from 6A-FL-overexpressing H1299 cells showed that the expression levels of 437 genes were 1.5 times greater compared to control cells (Fig. 2A). The 25 genes related to cell migration were selected by Ingenuity Pathway Analysis (IPA), and HMOX1 had the highest fold change among them: 18.1 × with a *p* value < 0.0026 (Fig. 2B).

### HMOX1 is the downstream regulator of SEMA6A in migration signaling in H1299 cells

**Figure 3:**
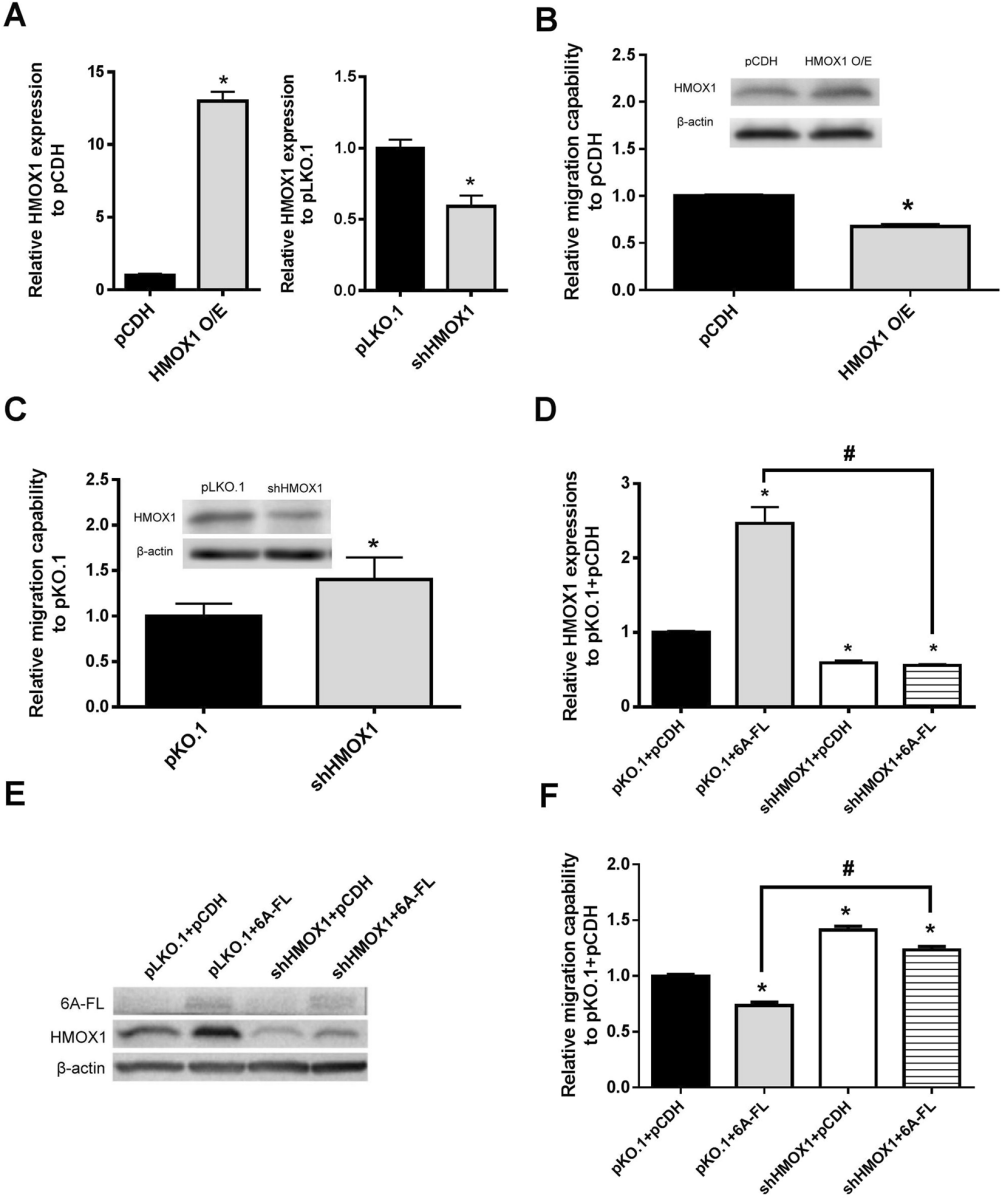
Expression of HMOX1 and migration capability by transwell assay of H1299 cells transfected with different vectors. (**A**) Relative mRNA expressions of HMOX1 in HMOX1 -overexpressing (HMOX1 O/E) (left) or -silenced (shHMOX1) (right) H1299 cells. (**B**) Migration capability of H1299 cells overexpressing HMOX1 or empty vector (pCDH). Inset: western blot of HMOX1. (**C**) Migration capability of HMOX1-silenced H1299 cells and cells containing empty vector (pLKO.1). Inset: western blot of HMOX1. (**D**) mRNA and (**E**) protein expression of HMOX1 in H1299 cells co-transfected with either pLKO.1 + pCDH, pLKO.1 + 6A-FL, shHMOX1 + pCDH, or shHMOX1 + 6A-FL. (F) Migration capability of H1299 cells co-transfected with either pLKO.1 + pCDH, pLKO.1 + 6A-FL, shHMOX1 + pCDH, or shHMOX1 + 6A-FL. *Statistical significance compared to empty vector-transfected H1299 cells at *p < *0.05, n = 3. # Statistical significance compared to each other at *p < *0.05, n = 3.

Since SEMA6A overexpression induced HMOX1 in H1299 cells, we hypothesized that SEMA6A could regulate HMOX1 to attenuate cell migration. To explore the effect of HMOX1 on migration, we evaluated the migration capability of HMOX1-overexpressing and -silenced H1299 cells using the transwell assay. The mRNA levels of HMOX1 were successfully regulated in the H1299 cells (Fig. 3A). The results of this assay showed that the migration rates were lower in HMOX1-overexpressing cells (Fig. 3B) and higher in HMOX1-silenced cells (Fig. 3C) compared to the respective control cells. Thus, HMOX1 plays a role in inhibition of migration in H1299 cells.

**Figure 4:**
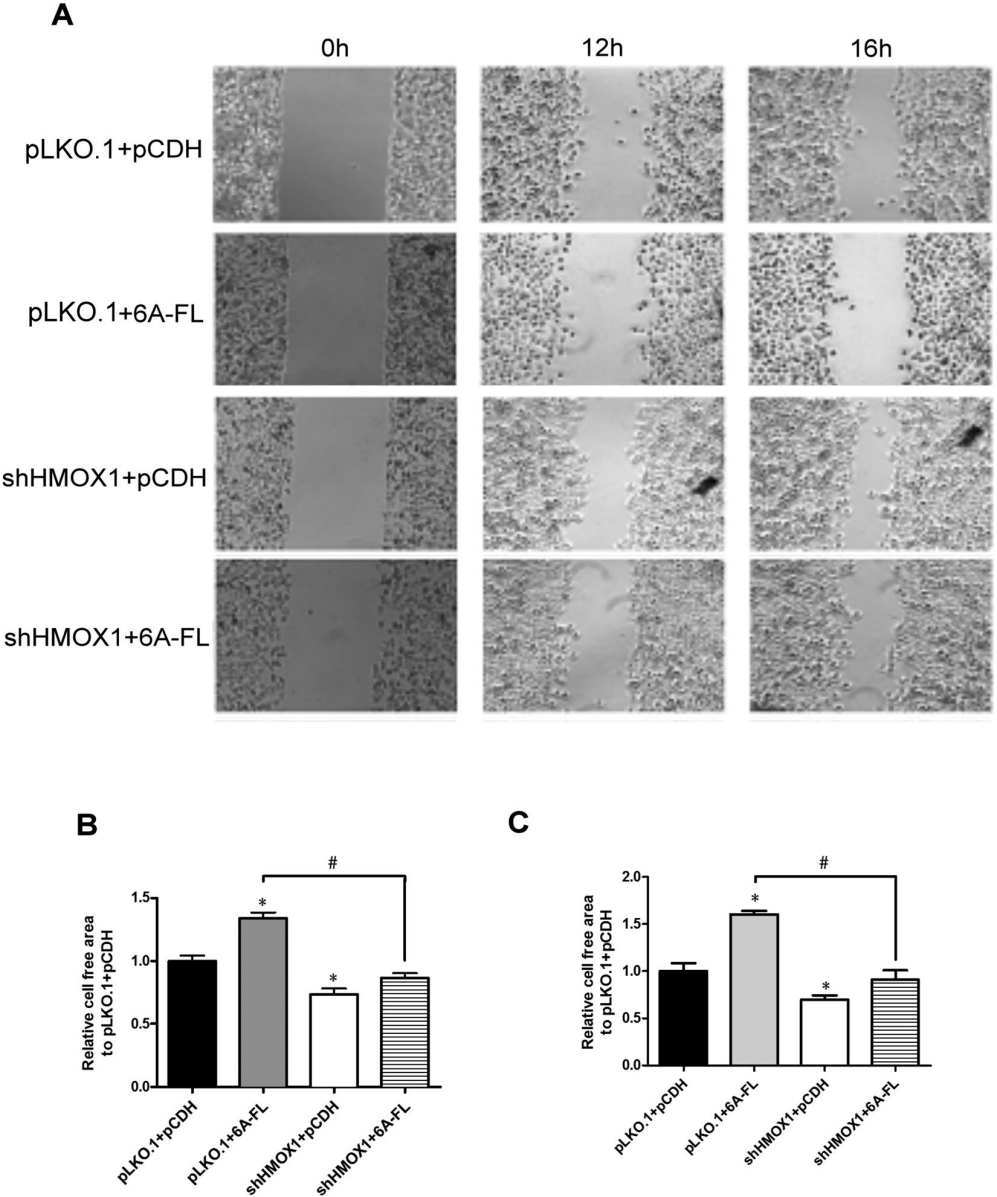
Migration capability evaluated by wound-healing assays. (**A**) Representative wound healing pictures at 0 h, 12 h, and 16 h. (**B**,**C)** Mobility rate histograms of each group at 12 h (**B**) and 16 h (**C**), expressed as the cell-free area. *Statistical significance compared to empty vectors (pLKO.1 + pCDH) at *p* < 0.05. ^#^Statistical significance compared to each other at *p* < 0.05.

Next, the involvement of HMOX1 in the action of SEMA6A in the H1299 cells was investigated by determining the migration capability of H1299 cells co-transfected with empty pLKO.1 and empty pCDH, empty pLKO.1 and 6A-FL, shHMOX1 and empty pCDH, or shHMOX1 and 6A-FL using transwell and wound-healing assays. The pLKO.1 + 6A-FL co-transfected H1299 cells had the highest expression levels of HMOX1, followed by the negative control H1299 cells (Fig. 3D,E). The levels of HMOX1 were the lowest in the shHMOX1 + pCDH cells and shHMOX1 + 6A-FL cells (Fig. 3D,E). The H1299 cells co-transfected with empty pLKO.1 and 6A-FL had a lower migration rate than the H1299 cells co-transfected with empty pLKO.1 and empty pCDH or with shHMOX1 and 6A-FL in transwell assay (Fig. 3F). Similarly, the cell-free area was larger in the empty pLKO.1 + 6A-FL cells than in the other co-transfected cells in the wound-healing assay (Fig. 4). These results suggest that silencing HMOX1 reverses the SEMA6A-derived attenuation of migration in H1299 cells.

### Nuclear Factor, Erythroid 2 Like 2 (NRF2) knockdown reverses the effect of SEMA6A on HMOX1 and migration in H1299 cells

**Figure 5:**
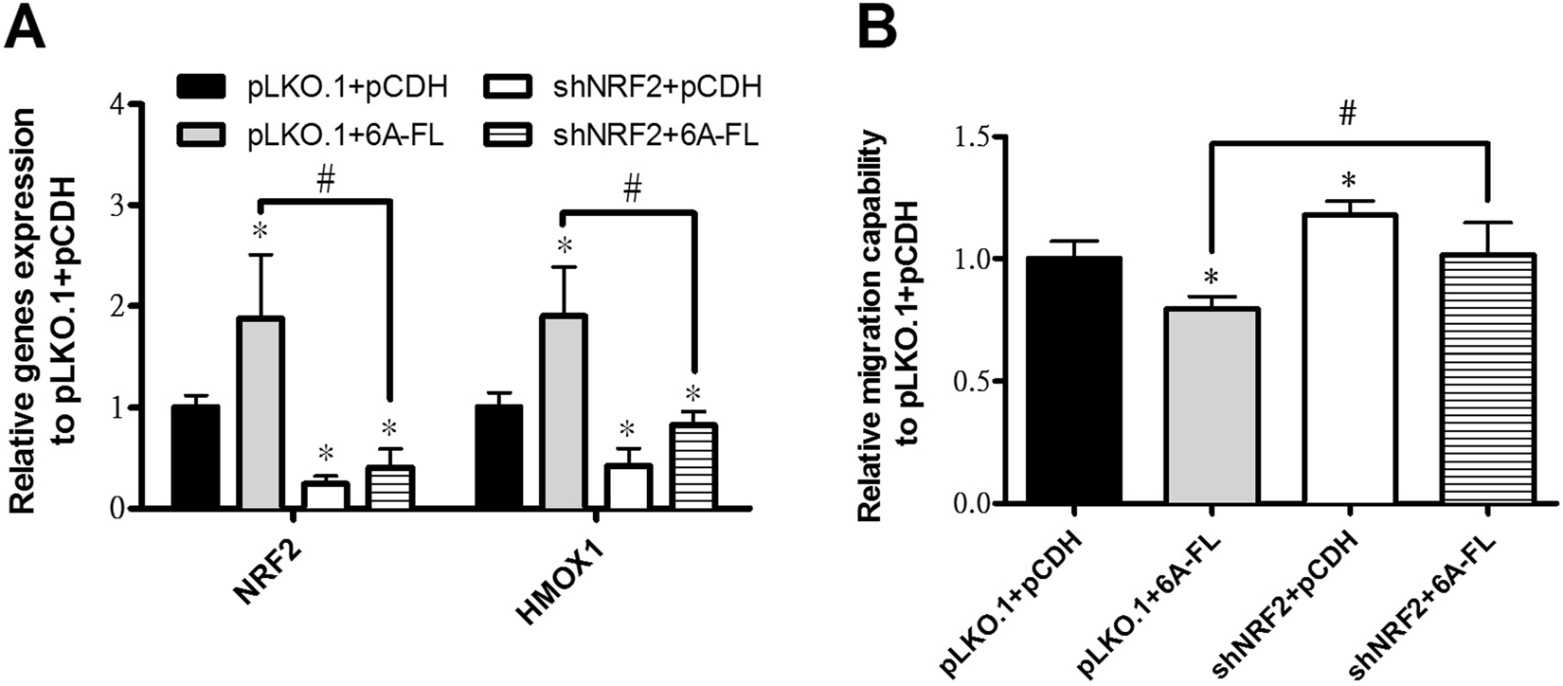
Effect of NRF2 on gene expression and migration capability of H1299 cells. (**A**) mRNA expression of NRF2 and HMOX1 and (**B**) migration capability in H1299 cells co-transfected with either pLKO.1 + pCDH, pLKO.1 + 6A-FL, shNRF2 + pCDH, or shNRF2 + 6A-FL. *Statistical significance compared to empty vectors (pLKO.1 + pCDH) at *p* < 0.05. ^#^Statistical significance compared to each other at *p* < 0.05.

NRF2 was reported as an upstream regulator of HMOX1 in another lung cancer cell line, H292^20^. Although NRF2 was not included in the first selection, the results of microarray showed that NRF2 was 1.3x up-regulated in SEMA6A-overexpressing H1299 cells. Moreover, 6A-FL-overexpressing H1299 cells had a significantly higher expression of NRF2 determined by RT-qPCR compared to control cells (Fig. 5A). Therefore, we also investigated if SEMA6A uses NRF2 to induce HMOX1, followed by inhibition of migration in H1299 cells. Similar to HMOX1 knockdown, NRF2 knockdown decreased expression of HMOX1 and increased the migration rate of H1299 cells (Fig. 5A,B, open bars). Moreover, it significantly lowered the expression of HMOX1 in 6A-FL-overexpressing H1299 cells, which was associated with an increase in migration rate (Fig. 5A,B, striped bars). These results indicate that NRF2 is an important upstream mediator of the action of HMOX1 in SEMA6A-induced attenuation of migration.

### SEMA6A regulates mRNA expression of Plasminogen Activator, Urokinase (PLAU), Insulin Like Growth Factor Binding Protein 3 (IGFBP3), Matrix metalloproteinase (MMP)-1, and MMP9 via HMOX1

**Figure 6:**
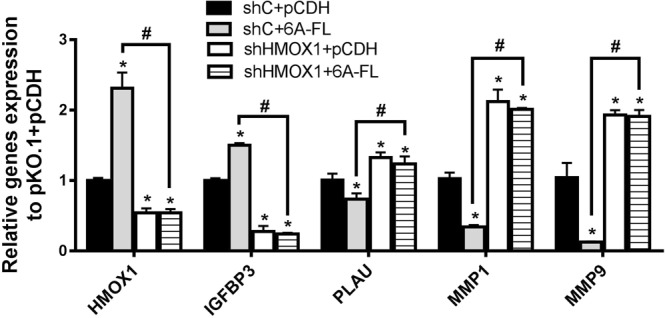
mRNA expression of IGFBP3, PLAU, MMP1, and MMP9 in H1299 cells. Cells were co-transfected with either pLKO.1 + pCDH, pLKO.1 + 6A-FL, shHMOX1 + pCDH, or shHMOX1 + 6A-FL. *Statistical significance compared to empty vectors (pLKO.1 + pCDH) at *p* < 0.05. ^#^Statistical significance compared to each other at *p* < 0.05.

For improving the understanding of SEMA6A-derived migration pathway, we also determined the mRNA expression levels of PLAU, MMP1 and MMP9 using RT-qPCR, because these genes were indicated to be reduced by HMOX1 and to induce migration in cancer cells^20–22^. Moreover, IGFBP3 was up-regulated by HMOX1, which attenuated migration capability of cancer cells^22^. The results showed that overexpression of 6A-FL decreased the mRNA levels of PLAU, MMP1, and MMP9 and increased the mRNA levels of IGFBP3 (Fig. 6). The 6A-FL-induced alterations were reversed when HMOX1 was silenced in 6A-FL-overexpressing H1299 cells.

## Discussion

Our data highlight a novel feature of SEMA6A, which is that SEMA6A can reduce lung cancer cell migration through the NRF2/HMOX1 axis in a manner dependent on its cytosolic region. The SEMA6A-derived regulation pathway in migration was also found to occur in other lung cancer cell lines, such as A549 and CL1-5 (Supplementary Fig. 1). Moreover, we demonstrated that SEMA6A employs HMOX1 to regulate the expression of genes related to cell migration, such as PLAU, MMP1, MMP9, and IGFBP3. This newly discovered function and mechanism of SEMA6A indicates that SEMA6A may be a cancer suppressor and can be a biomarker of lung cancer.

Increased cell migration is a characteristic of cancer recurrence and results in low efficacy of cancer treatment^2,3^. Since low levels of SEMA6A are accompanied by high recurrence of cancer^19^, we hypothesized that SEMA6A plays a role in cancer cell migration. SEMA6A was initially described as a regulator of migration in neurons, granule cells, and endothelial cells^10,23–25^. Although overexpression of the extracellular region of SEMA6A in cancer cells suppressed the migration of vascular endothelial cells, the effect of SEMA6A on the migration of cancer cells themselves is still unknown^10^. In the present study, we demonstrated that SEMA6A plays a role in suppressing migration in H1299 cells, which had a lower migration rate when overexpressing 6A-FL, but a higher migration rate when SEMA6A was silenced. Since some semaphorins, such as SEMA6D and SEMA1A, have been reported to function as both receptors and ligands, we further investigated the possibility of SEMA6A reducing cell migration via reverse or forward signaling. Reverse but not forward signaling involves a membrane protein transducing intracellular signals through its cytoplasmic domain(s), and the fact that the SEMA6A’s ectodomain construct had no effect on migration capability suggests that SEMA6A-derived migration suppression may be not through forward signaling but reverse signaling, due to its dependence on its cytosolic region.

Our results also demonstrated that SEMA6A modulated migration by inducing the NRF2/HMOX1 axis in H1299 cells. In most types of lung tumors, high intratumoral levels of NRF2 and HMOX1 are accompanied by poor clinical outcomes^26,27^. Moreover, Tertil *et al*. (2015) reported that inducing the NRF2/HMOX1 axis could significantly decrease the migration capability of lung cancer cells^20^. NRF2 has been considered a tumor suppressor because *Nrf2*-deficient mice are more susceptible to carcinogens^28,29^, and the loss of *Nrf2* enhances the migration of cancer cells^20,30,31^. NRF2 is regulated by several genes and pathways at the transcriptional and post-transcriptional levels^32^. In light of the observed increase of NRF2 mRNA upon overexpression of 6A-FL, SEMA6A probably regulates NRF2 at the transcriptional level. The aryl hydrocarbon receptor (AhR) and aryl hydrocarbon receptor nuclear translocator (ARNT) complex is one of the transcription factors of NRF2, and the activity of this complex has been suggested to reduce cancer metastasis through increasing the expression of NRF2^32–34^. Therefore, SEMA6A might induce NRF2 by regulating either activation or expression of AhR and ARNT in H1299 cells.

HMOX1 was reported as a downstream effector of NRF2 in the migration suppression pathway in lung cancer cells^20^. Traditionally, HMOX1 is known as an enzyme that maintains cellular homeostasis under stress^35^. However, there are more and more studies revealing the functional role of HMOX1 in cancer. For example, overexpression of HMOX1 in lung cancer cells attenuated the expression of matrix metalloproteinases (MMPs), which enhance the migration of cancer cells^20,36^. In the present study, we also demonstrated that HMOX1 was employed by SEMA6A to reduce MMP1 and MMP9 (Fig. 6). In addition, HMOX1 up-regulated E-cadherin and β-catenin expression, increased filopodia zippering at the leading edge of cells, and favored a more epithelial phenotype^37,38^. Recently, RNA-seq data from prostate cancer cells overexpressing HMOX1 showed that HMOX1 down-modulated the PLAU pathway related to cell adhesion and cell-cell communication. They presented that HMOX1 reduced PLAU directly affecting Rho GTPases through the alpha V-Beta 3 integrin receptor, which in turn increased the potentiality of tumor cells to adhere to each other^38^. Moreover, to silence PLAU led to a reduced capability of migration and invasion in lung cancer cell lines, H1299 and A549^21^. Since the expression of PLAU was regulated by HMOX1 in 6A-FL-overexpressing H1299, PLAU might also be involved in the SEMA6A-induced pathway of migration suppression.

IGFBP3 was another gene induced in 6A-FL-overexpressing cells. Overexpression of IGFBP3 reduced migration and invasion by down-regulating PLAU in lung cancer cell lines H1299 and A549^22^. Torng *et al*. (2008) showed that IGFBP3 was one of the significantly suppressed genes in endometrioid carcinoma cells with high invasion^39^. Moreover, low expression of IGFBP3 correlated clinically with higher tumor grade, advanced stage, and poor survival^39,40^. In our study, 6A-FL overexpression induced IGFBP3, while the induction of IGFBP3 was reversed if HMOX1 was silenced in the 6A-FL-overexpressing cells. Therefore, IGFBP3 is another potential candidate involved in the SEMA6A-induced migration suppression pathway.

In conclusion, the present study identified a novel suppressor of lung cancer cell migration, SEMA6A, which attenuates migration by inducing the NRF2/HMOX1 axis. Our results not only explain why low SEMA6A expression correlates with high recurrence rates in clinical trials, but also suggest that SEMA6A could be exploited in cancer therapy.

## Materials and Methods

### Cell culture

The HEK-293T cell line was provided by Dr. Shau-Ping Lin (Institute of Biotechnology, National Taiwan University) and cultured in DMEM (GIBCO, CA, USA), and the H1299 cell line was purchased from Bioresource Collection and Research Center (Hsinchu, Taiwan) and cultured in RPMI-1640 (GIBCO, CA, USA). All cells were incubated with medium containing 10% fetal bovine serum (FBS) and 1% penicillin/streptomycin at 37 °C in a 5% CO_2_ humidified incubator.

### RNA extraction and reverse transcription

Total RNA was extracted from cells using an RNeasy Mini kit (Qiagen, Hilden, Germany), and 1 μg of RNA from each sample was reverse-transcribed using a High-Capacity cDNA Reverse Transcription kit (Applied Biosystems, Foster City, CA, USA), according to the instructions of the manufacturer.

### Gene constructs and shRNA

6A-FL and HMOX1 were generated from Hs68 and H1299 cells, respectively, using RT-PCR. 6Aect was produced from the 6A-FL sequence. 6A-FL and 6Aect were given a 6xHis tag sequence at the C-terminus. The primers for these constructs are listed in Supplementary Table 1. The shRNAs for SEMA6A (TRCN0000061108 and TRCN0000061109), HMOX1 (TRCN0000290436), and NRF2 (TRCN0000007558) were purchased from the RNAi Core Facility (Academia Sinica, Taipei, Taiwan).

### Virus production and cell infection

HEK-293T cells (4 × 10^6^ cells) were co-transfected with packaging plasmid (psPAX2; 6 μg), envelope plasmid (pMD2G; 2 μg), and the transfer vector plasmid (pCDH-CMV-MCS-EF1-puro for overexpression; pLKO.1 for gene silence; 8 μg), which was empty or contained either target genes or shRNA, using TransIT®-2020 Transfection Reagent (Mirus Bio LLC). The supernatants were collected at 48 and 72 h post-transfection and stored at −80 °C. H1299 cells were seeded in 24-well plates at 4 × 10^4^ cells/well and incubated for 24 h, and then 1 mL of viral supernatant was added into each well and centrifuged at 2500 rpm for 60 min at room temperature followed by incubation at 37 °C in 5% CO_2._

### Western blotting

For protein extraction, cells were lysed by RIPA lysis buffer (Millipore, CA, USA) containing protease inhibitor cocktail, 10 mM β-glycerophosphate, and 5 mM Na_3_VO_4_ followed by sonicating homogenization for 20 s on ice. The protein concentration was measured by the Bradford protein assay (Bio-Rad, Hercules, CA, USA). Equal amounts of protein from each sample were separated by 10% SDS-PAGE and then transferred onto a nitrocellulose membrane (Millipore). After incubation overnight at 4 °C with primary antibodies against 6 × His (Millipore), SEMA6A (Genetex, Hsinchu,Taiwan), HMOX1 (Millipore), or β-actin (Millipore), the samples were hybridized with horseradish peroxidase (HRP)-conjugated secondary antibodies at room temperature for 1 h. Finally, HRP activity was visualized by an enhanced chemiluminescence system (UVP BioSpectrum Imaging System).

### Transwell assay

The 6.5 mm-diameter cell culture inserts (8 μm pore size) were used to perform transwell assays in 24-well plates. After starving the cells with serum-free medium for 24 h, they were seeded at 5 × 10^4^ cells/well in the upper chamber of the filter in 200 μL of FBS-free medium. Then, 0.75 mL of complete medium containing 10% FBS was added in the lower chamber. After 16 h, cells were fixed for 15 min at room temperature with 10% acetic acid containing 10% methanol, and washed with 1× PBS. The non-migrating cells on the inner transwell membrane were wiped carefully, and the membranes were stained with 1 mL of 0.5% crystal violet for 30 min and washed with water. The stained cells were solubilized with 10% acetic acid and quantitated on a microplate reader at 580 nm.

### Microarray data analysis

Microarray data were from our previous unpublished studies, and the intensity data of *SEMA6A* were analyzed using Partek software (Partek, Chesterfield, MO, USA) to obtain mRNA expression levels. Robust multi-array average analysis was used to preprocess probe-level expression data of *SEMA6A*, including background correction, quantile normalization, and summarization. The genes were selected when their fold changes were >1.5 and p values were <0.01 using paired t-tests. Functional grouping and pathway analysis were performed using IPA software (Ingenuity Systems, Redwood City, CA, USA).

### Quantitative PCR

Quantitative PCR was processed using an ABI 7300 instrument (Applied Biosystems, Foster City, CA, USA) with a SYBR Green Master Mix kit (Applied Biosystems) according to standard procedures. The sequences of the gene-specific primers (Purigo, Taipei, Taiwan) are listed in Supplementary Table 1. GAPDH was used as an internal control for normalizing the mRNA levels of tested genes.

### Wound-healing assay

Approximately equal amount of cells were loaded into the left and right chambers of an ibidi Culture-Insert (Applied BioPhysics, NY, NY, USA). Each insert was placed in one well of a 24-well plate. The experiments were carried out in triplicate. After overnight incubation at 37 °C and 5% CO_2_, the insert was removed and a photo of each well was taken at 0, 12, and 16 h. The cell-free area was measured by ImageJ software at every time point.

### Statistical analysis

A two-tailed Student’s t-test was applied to all the data in this study. Differences were considered to be significant if *p* < 0.05. All values in the study are presented as mean ± standard error of mean of three or more experiments.

## Supplementary information

Supplementary materials
