# Supplementary material for: Semaphorin 6A Attenuates the Migration Capability of Lung Cancer Cells via the NRF2/HMOX1 Axis
Source: Sci Rep. 2019 Sep 16;9:13302. doi: 10.1038/s41598-019-49874-8 (PMC6746772; doi:10.1038/s41598-019-49874-8)
Supplement: Supplementary file 1 — Supplementary materials [file 41598_2019_49874_MOESM1_ESM.pdf]

Semaphorin 6A Attenuates the Migration Capability of Lung Cancer Cells via the NRF2/HMOX1 Axis

Li-Han Chen[1], Che-Yu Liao[2], Liang-Chuan Lai[3, 4], Mong-Hsun Tsai[2, 4, 5, 6, 7, *], and Eric Y. Chuang[1, 6, 8, 9, *]

[1] Graduate Institute of Biomedical Electronics and Bioinformatics, National Taiwan University, Taipei, Taiwan.

[2] Institute of Biotechnology, National Taiwan University, Taipei, Taiwan

[3] Institute of Physiology, National Taiwan University, Taipei, Taiwan

[4] Genome and Systems Biology Degree Program, National Taiwan University, Taipei, Taiwan

[5] Center for Biotechnology, National Taiwan University, Taipei, Taiwan.

[6] Bioinformatics and Biostatistics Core, Center of Genomic Medicine, National Taiwan University, Taipei, Taiwan.

[7] Agricultural Biotechnology Research Center, Academia Sinica, Taipei, Taiwan

[8] School of Chinese Medicine, China Medical University, Taichung, Taiwan.

[9] Biomedical Technology and Device Research Laboratories, Industrial Technology Research Institute, Hsinchu, Taiwan.

* Correspondence:

Dr. Eric Y. Chuang, Graduate Institute of Biomedical Electronics and Bioinformatics, National Taiwan University, MD-622, No. 1, Section 4, Roosevelt Rd., Taipei City 10617, Taiwan, E-mail: chuangey@ntu.edu.tw, Tel: +886-2-33663660.

Dr. Mong-Hsun Tsai, Institute of Biotechnology, National Taiwan University, No. 1, Section 4, Roosevelt Rd., Taipei City 10617, Taiwan, E-mail: motiont@gmail.com, Tel: +886-2-33666009

Supplementary Figure Legends

Supplementary figure 1. Effect of SEMA6A overexpression in lung cancer cell lines. (A) Migration capability of 6A-FL-overexpressing A549 and CL1-5 cells. Expressions of HMOX1 and NRF2 in 6A-FL-overexpressing A549 (B) and CL1-5 (C) cells. * Statistical significance compared to empty vector-transfected H1299 cells at $p < 0.05$, n = 3.

Supplementary figure 2.
Whole blot membrane scans used for preparing (A) Fig. 1A left, (B) Fig. 1A right, (C) Fig. 1B, (D) Fig. 3B, (E) Fig. 3C, and (F) Fig. 3E in this study.

Supplementary table 1. Primer list

Gene ID	NM ID	primers
6A-FL		5’-ATGAGGTCAGAAGCCTTGCT-3’<br>5’-TGTACACGCATCATTG-3’
6Aect		5’-ATTCCTTTGTGGCACTGAATGGGC-3’<br>5’-TGCTGTCAGGTGAGTCAAGCAGAT-3’
SEMA6A	NM_020796	5’-ATTCCTTTGTGGCACTGAATGGGC-3’<br>5’-TGCTGTCAGGTGAGTCAAGCAGAT-3’
GAPDH	NM_002046	5’-CTTTGGTATCGTGGAAGGAC-3’<br>5’-TAGAGGCAGGGATGATGTT-3’
HMOX1	NM_002133	5’-ACCAAGTTCAAGCAGCTCTAC-3’<br>5’-GCAGTCTTGGCCTCTTCTATC-3’
NRF2	NM_006164	5’-TGATTCTGACTCCGGCATTT-3’<br>5’-GCCAAGTAGTGTGTCTCCATAG-3’
IGFBP3	NM_001013398	5’-CAGAGCACAGATACCCAGAAC-3’<br>5’-GGACTCAGCACATTGAGGAA-3’
PLAU	NM_002658	5’-GACAGTGCCTGGGAATGTATTA-3’<br>5’-AGGGATAACTGGCCAAGAAAG-3’

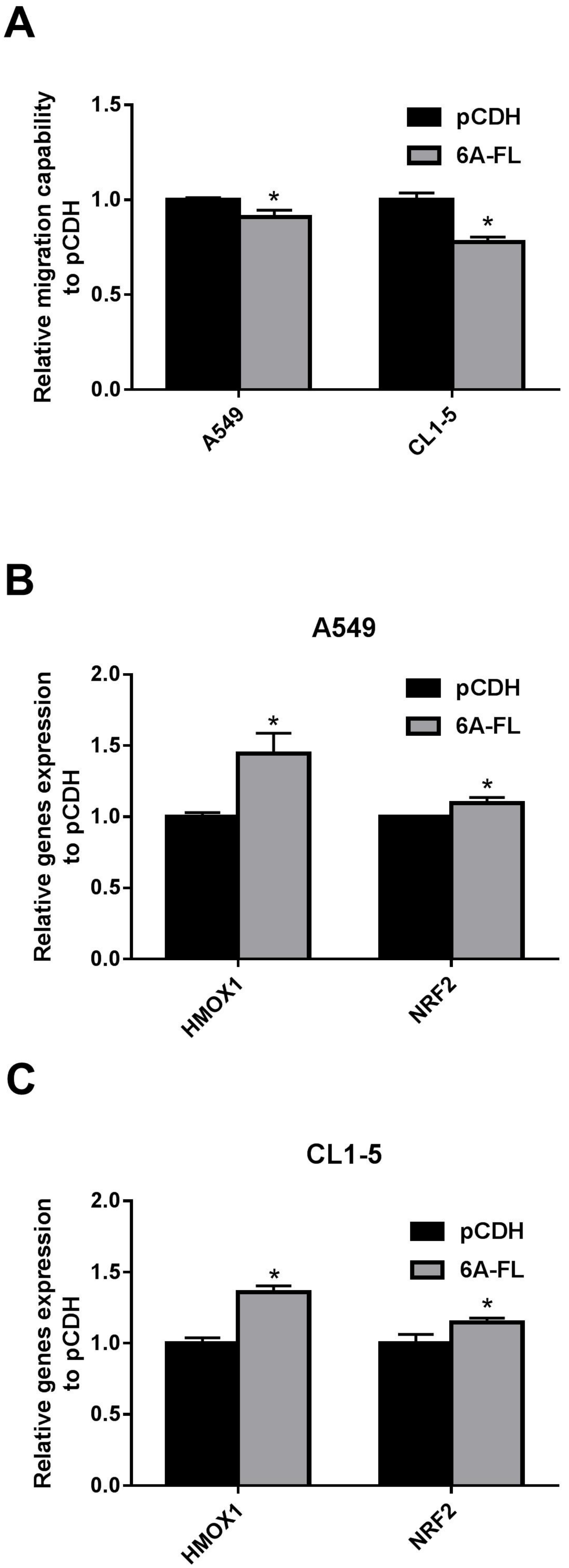

Supplementary figure 1

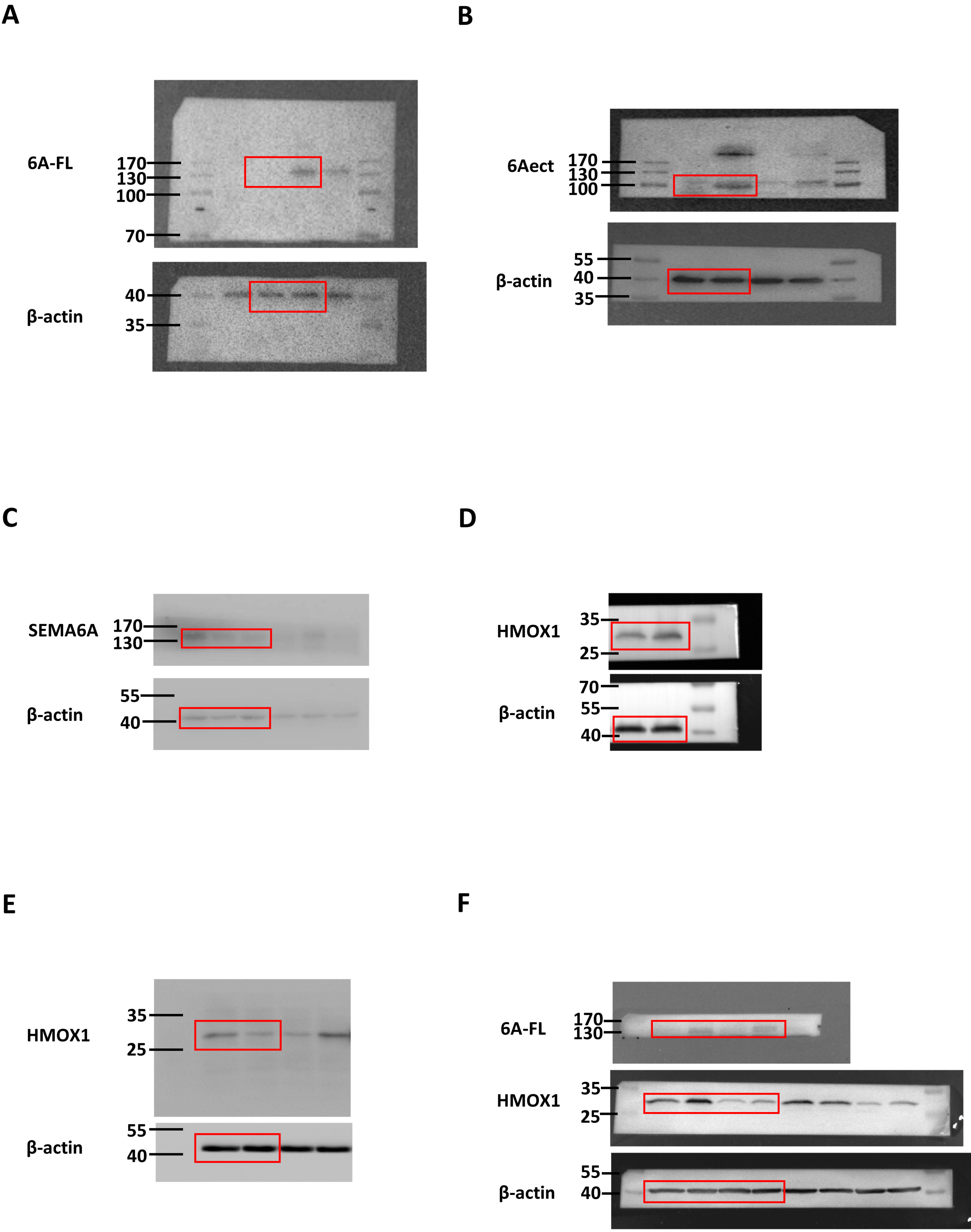

Supplementary figure 2
